# Intercontinental Spread of Eurasian Highly Pathogenic Avian Influenza A(H5N1) to Senegal

**DOI:** 10.3201/eid2801.211401

**Published:** 2022-01

**Authors:** Fatou T. Lo, Bianca Zecchin, Alpha A. Diallo, O. Racky, Luca Tassoni, Aida Diop, Moussa Diouf, Mayékor Diouf, Yacine N. Samb, Ambra Pastori, Federica Gobbo, Francesca Ellero, Mariame Diop, Modou M. Lo, Mame N. Diouf, Mathioro Fall, Amadou A. Ndiaye, Adji M. Gaye, Médoune Badiane, Mbargou Lo, Babacor N. Youm, Ibrahima Ndao, Marius Niaga, Calogero Terregino, Boly Diop, Youssou Ndiaye, Angelique Angot, Ismaila Seck, Mamadou Niang, Baba Soumare, Alice Fusaro, Isabella Monne

**Affiliations:** Institut Sénégalais de Recherches Agricoles–Laboratoire National de l’Elevage et de Recherches Vétérinaires, Dakar-Hann, Senegal (F.T. Lo, A.A. Diallo, R.O. Ba, A. Diop, Moussa Diouf, Mayékor Diouf, Y.N. Samb, M. Diop, M.M. Lo, M.N. Diouf);; Istituto Zooprofilattico Sperimentale delle Venezie, Legnaro, Italy (B. Zecchin, L. Tassoni, A. Pastori, F. Gobbo, F. Ellero, C. Terregino, A. Fusaro, I. Monne);; Direction des Services Vétérinaires, Rufisque, Senegal (M. Fall, A.A. Ndiaye, A.M. Gaye, M. Badiane, M. Lo);; Direction des Parcs Nationaux, Dakar, Senegal (B.N. Youm, I. Ndao, M. Niaga);; Direction de la Prévention, Dakar (B. Diop);; Food and Agriculture Organization of the United Nations, Dakar (Y. Ndiaye);; Food and Agriculture Organization of the United Nations, Rome, Italy (A. Angot);; Food and Agriculture Organization of the United Nations, Accra, Ghana (I. Seck, M. Niang, B. Soumare)

**Keywords:** influenza, HPAI H5N1 virus, Senegal, poultry, white pelicans, transcontinental spread, zoonoses, viruses

## Abstract

In January 2021, Senegal reported the emergence of highly pathogenic avian influenza virus A(H5N1), which was detected on a poultry farm in Thies, Senegal, and in great white pelicans in the Djoudj National Bird Sanctuary. We report evidence of new transcontinental spread of H5N1 from Europe toward Africa.

On December 23, 2020, a single poultry farm composed of 4 barns of laying hens having a total of 102,000 birds in Pout, Thies Region, Senegal, reported increased deaths (mortality rate 58%) to animal health authorities. The clinical signs observed in the affected poultry were edema of the cervical region, cyanosis, congestion of the crests and barbs, and a state of general prostration. Organs and cloacal and oropharyngeal swab specimens collected from dead and sick birds were analyzed at the National Veterinary Laboratory for Livestock and Research (LNERV; Dakar, Senegal), where highly pathogenic avian influenza (HPAI) A (H5N1) virus was confirmed on January 7, 2021. 

Later that month, 750 great white pelicans (*Pelecanus onocrotalus*) (740 juveniles and 10 adults) were found dead by rangers in the Djoudj National Bird Sanctuary, a UNESCO World Heritage site, which is a wetland near the Senegal–Mauritania border. The sanctuary welcomes thousands of Palearctic and Afrotropical migratory birds every year as a refuge, feeding site, and breeding site. On January 15, 2021, the monthly count of birds at Djoudj enacted by the Ministry of Environment documented 8,887 pelicans, for an estimated mortality rate of 8.4% in January 2021. After identifying H5N1 in the dead birds, LNERV analyzed amino acid sequences deduced at the hemagglutinin cleavage site (PLREKRRKR×GLF) on samples from poultry and pelicans, which classified the strain as an HPAI.

Since the emergence of the HPAI H5Nx viruses of the goose/Guangdong (gs/Gd) lineage in 1996, the transcontinental spread of the virus to Africa has been described at least 3 times (*1*). According to available data, no incursions have involved Senegal before. This unprecedented geographic spread raises questions about the mechanisms of emergence and dissemination of HPAI H5N1 in this country. To determine the origin and transmission pathways of the virus, we analyzed the complete genome of 4 HPAI H5N1 viruses collected in Senegal from domestic and wild birds, studying their spatial diffusion dynamics.

## The Study

A total of 8 clinical samples were submitted to the World Organisation for Animal Health Reference Laboratory and to the Food and Agriculture Organization of the United Nations Reference Center for Avian Influenza and Newcastle Disease at the Istituto Zooprofilattico Sperimentale delle Venezie (Legnaro, Padova, Italy) for confirmatory diagnosis and genetic characterization of the identified viruses. HPAI H5N1 was identified in all submitted samples by molecular analysis, confirming the results from LNERV. Because of the low viral load, whole-genome sequences were successfully generated from only 4 of 8 samples (Table) collected from poultry and wild birds, as previously described (*1*).

**Table:** HPAI H5N1 viruses identified from chicken and pelican samples, Senegal, 2020–2021*

Sample type	Virus name	Species	Location	Latitude and longitude	Collection date	GISAID accession no.
Cloacal swab	A/chicken/Senegal/21VIR1084–3/2021	Chicken	Thies region	14.781388, 17.042222	2020 Dec 23	EPI1866442–9
Cloacal swab	A/chicken/Senegal/21VIR1084–4/2021	Chicken	Thies region	14.781388, 17.042222	2020 Dec 23	EPI1866450–7
Cloacal swab	A/chicken/Senegal/21VIR1084–5/2021	Chicken	Thies region	14.781388, 17.042222	2020 Dec 23	EPI1866458–65
Oropharyngeal swab	A/great-white_pelican/Senegal/21–67_21VIR1084–8/2021	Great white pelican	Djoudj National Bird Sanctuary	16.352169, 16.277897	2021 Jan 23	EPI1866466–73

*Sequences were submitted to GISAID's EpiFlu database (https://www.gisaid.org). HPAI, highly pathogenic avian influenza.

The phylogenetic analysis of the hemagglutinin gene revealed that the 4 HPAI H5N1 viruses from Senegal belong to clade 2.3.4.4b and cluster not only together but also with the HPAI H5N1 viruses that have been circulating in Europe since October 2020 (99.8%–99.9% nucleotide similarity) (Appendix Figure 1) (*2*,*3*). In particular, the HPAI H5N1 viruses from Senegal cluster together in the phylogenetic trees of all 8 gene segments and are closely related to HPAI H5N1 viruses identified in the Netherlands, United Kingdom, and Italy during October–December 2020 (98.8%–100% nucleotide similarity) (Appendix Figures 1–8). These findings suggest virus introduction in the country was likely caused by wild birds’ migration routes from Europe.

To reconstruct the spatial spread and estimate time of virus introduction into Senegal, we performed a phylogeographic analysis of the hemagglutinin gene in BEAST version 1.10.4 (*4*). We defined 5 discrete geographic regions: Central Asia, Northern Europe, Eastern Europe, Southern Europe, and Senegal. The mean time to the most recent common ancestor of the HPAI H5N1 viruses from Senegal was estimated to be November 2020 (95% HPD interval October–December 2020). The genetic spatial analysis indicated that the virus had spread from Southern Europe to Senegal, which suggests that West Africa likely acted as the ecologic sink of the HPAI H5N1 viruses circulating in Europe (Figure; Appendix Figure 9). Because availability of viral sequences from different countries could affect phylogeographic analyses, having a large number of sequences available is vital to obtain accurate and reliable results.

**Figure:**
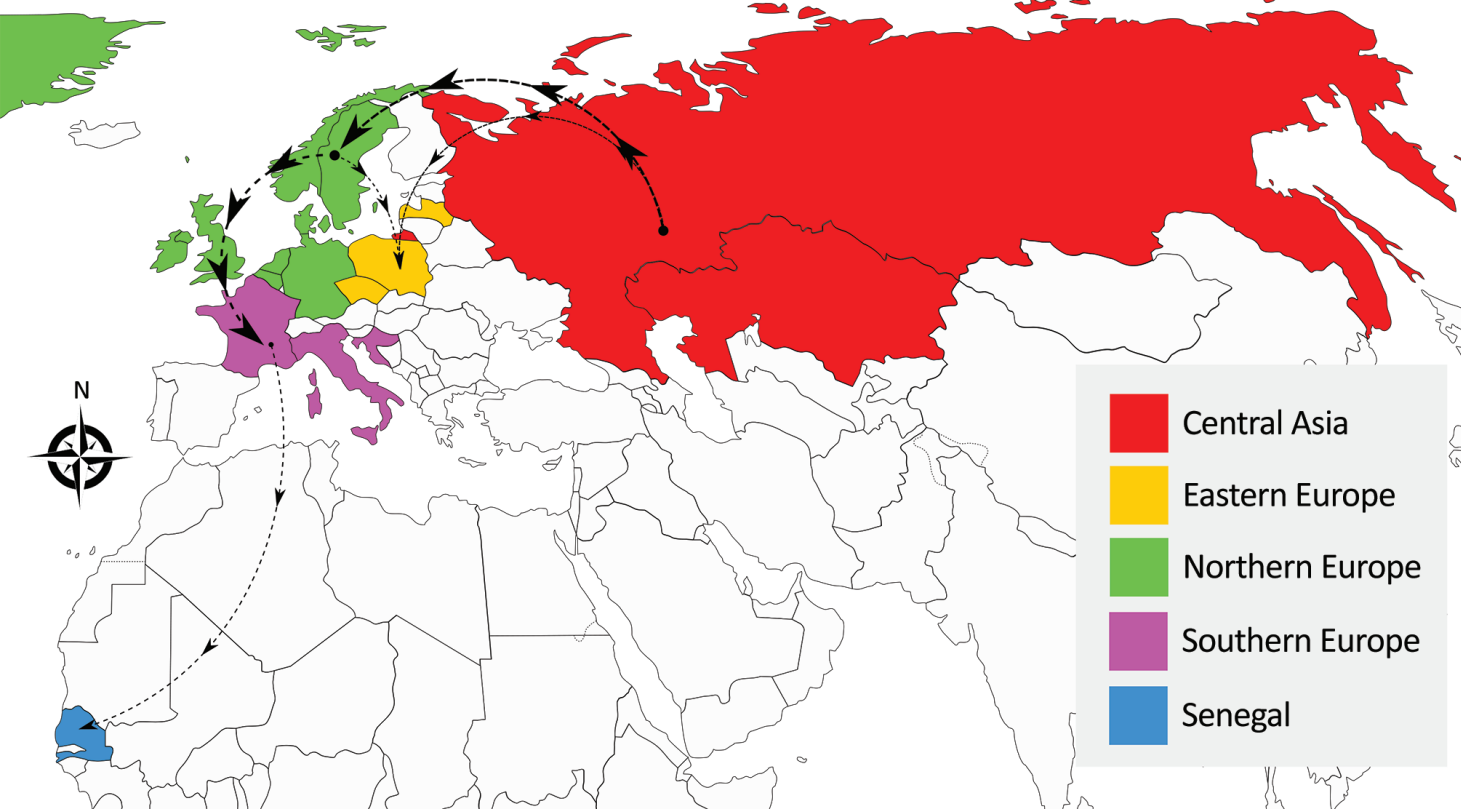
Origin and spread of the highly pathogenic avian influena H5N1 virus. Five discrete geographic regions, namely Central Asia, Northern Europe, Eastern Europe, Southern Europe, and Senegal, are defined and marked with different colors. The routes of migration supported by a Bayes factor >10 are displayed in the map as thin dashed lines; thicker dashed lines indicate Bayes factor >20.

## Conclusions

These evolutionary and spatial investigations indicate that the H5N1 outbreaks in Senegal did not emerge from local evolution of H5N1 viruses in Africa. These new viruses seem to have been introduced in fall 2020 from Eurasia through migratory birds flying southwest for winter. The estimated time to the most recent common ancestor (October–December 2020) and the long branches that separate the Senegal viruses from progenitors in Europe suggest an undetected virus circulating in the area, likely in wild birds. 

The Djoudj National Bird Sanctuary, located in the Senegal River delta along the East Atlantic Flyway, is a sanctuary for large breeding waterbirds, including great white pelicans. H5N1 caused the death of hundreds of pelicans there. Before the emergence of the HPAI H5Nx viruses of the Gs/GD lineage, infection with avian influenza virus of pelicans was rarely reported (*5*). The incursion of the Gs/GD lineage has resulted in numerous fatal infections in this species. On the basis of data from the avian influenza passive surveillance system implemented in Europe during 2005–2017, an HPAI detection rate of 9.5% has been estimated in great white pelicans (*6*). This species is highly gregarious, behavior that could have promoted the spread of HPAI in these birds in Senegal. Unfortunately, surveillance of wild and domestic birds near where the H5N1-infected pelicans were identified did not shed light on the species responsible for introducing the virus. The wetlands of Senegal are inhabited by millions of aquatic bird species, including Garganey (*Anas querquedula*), Northern pintail (*Anas acuta*), Northern shoveler (*Spatula clypeata*), Eurasian teal (*Anas crecca*), Eurasian wigeon (*Mareca penelope*), Common pochard (*Aythya farina*), and Tufted duck (*Aythya fuligula*), many of which have had a role in the spread of Gs/GD-lineage H5 viruses (*7*,*8*). This bird population has been hardly affected by the 2020–2021 epidemic in Europe, during which HPAI H5 virus infections were reported in apparently healthy birds (*2*). Therefore, even considering that most of the great white pelicans residing in the Djoudj National Bird Sanctuary are deemed to be sedentary (*9*), species other than pelicans might have been involved in introducing and spreading H5N1 within Senegal or to neighboring countries reporting HPAI H5N1, including Niger, Nigeria, Mauritania, and Mali. 

Recent reports of H5N1 in Senegal, Mauritania, and Mali indicate an unprecedented westward spread of the virus in Africa (*10*). However, the lack of genetic information on the viruses detected in these countries makes it difficult to reconstruct the exact number of virus introductions and dynamics of virus dissemination, and both poultry trade and wild bird movements remain valid candidate pathways. Moreover, after the outbreaks of HPAI in poultry, 2 states in Nigeria reported 7 suspected human cases of avian influenza H5N1, 4 in Kano and 3 in Plateau. These cases confirm the importance of One Health joint activities by public human and animal health sectors to contain and monitor virus spread and the emergence of novel viruses of major concern (*11*).

There is still much to learn about the ecology of these viruses in the wild bird population; detection of the 2.3.4.4b clade in Senegal demonstrates that predicting the dissemination trajectories of these viruses is difficult. No system yet exists that can prevent the virus from following wild bird movements. Efforts are needed to regulate poultry movements and develop risk-based surveillance in wild birds in Africa to detect newly introduced and circulating viruses, reduce the likely spread to poultry, and limit the risk for exposure of humans to infected birds.

Appendix Additional information about the intercontinental spread of Eurasian highly pathogenic avian influenza H5N1 to Senegal.
