# Supplementary material for: Intercontinental Spread of Eurasian Highly Pathogenic Avian Influenza A(H5N1) to Senegal
Source: Emerg Infect Dis. 2022 Jan;28(1):234–7. doi: 10.3201/eid2801.211401 (PMC8714199; doi:10.3201/eid2801.211401)
Supplement: Appendix — Additional information about the intercontinental spread of Eurasian highly pathogenic avian influenza H5N1 to Senegal. [file 21-1401-Techapp-s1.pdf]

Article DOI: https://doi.org/10.3201/eid2801.211401

Intercontinental Spread of Eurasian Highly Pathogenic Avian Influenza H5N1 to Senegal

Appendix

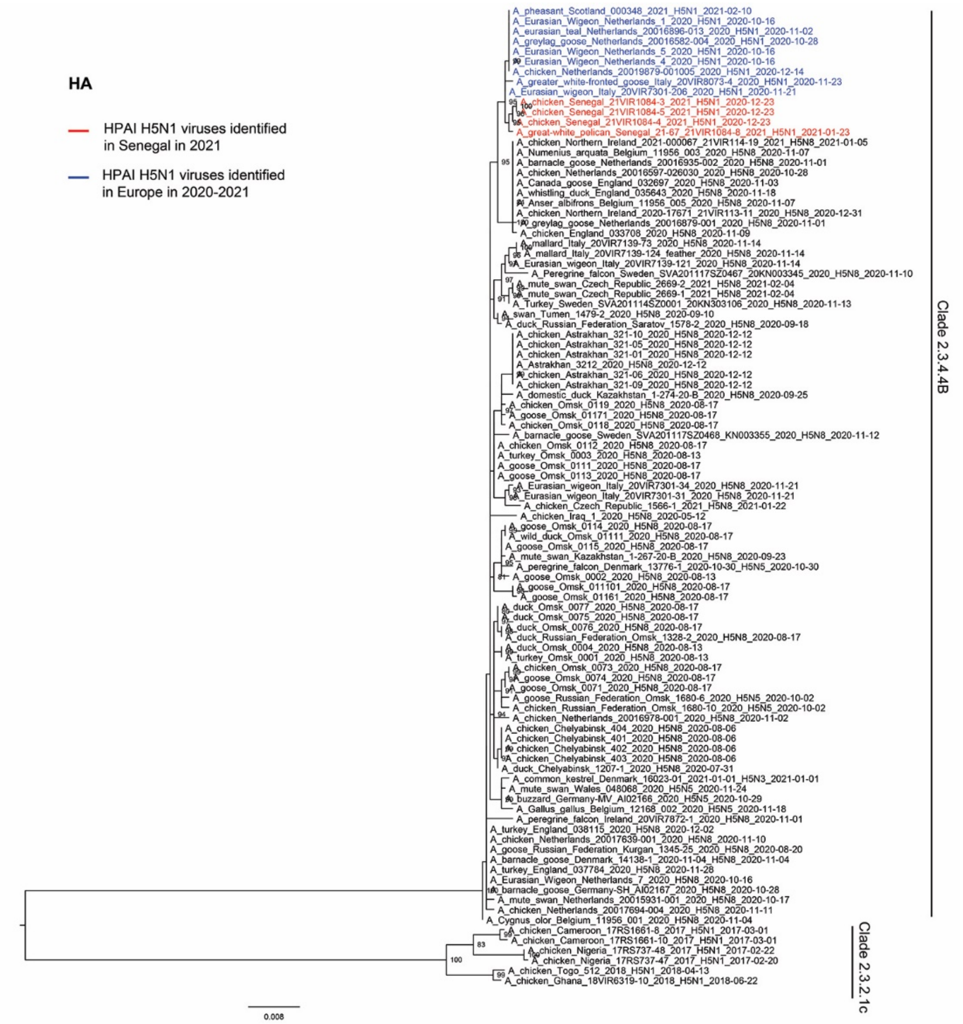

Appendix Figure 1. Maximum likelihood phylogenetic tree of the hemagglutinin (HA) gene (clade 2.3.4.4b) obtained by using IQTREE version 1.6.6. The HPAI H5N1 viruses from Senegal are marked in red; the HPAI H5N1 viruses from Europe are marked in blue. Ultrafast bootstrap supports >80 are indicated next to the nodes. HPAI, highly pathogenic avian influenza.

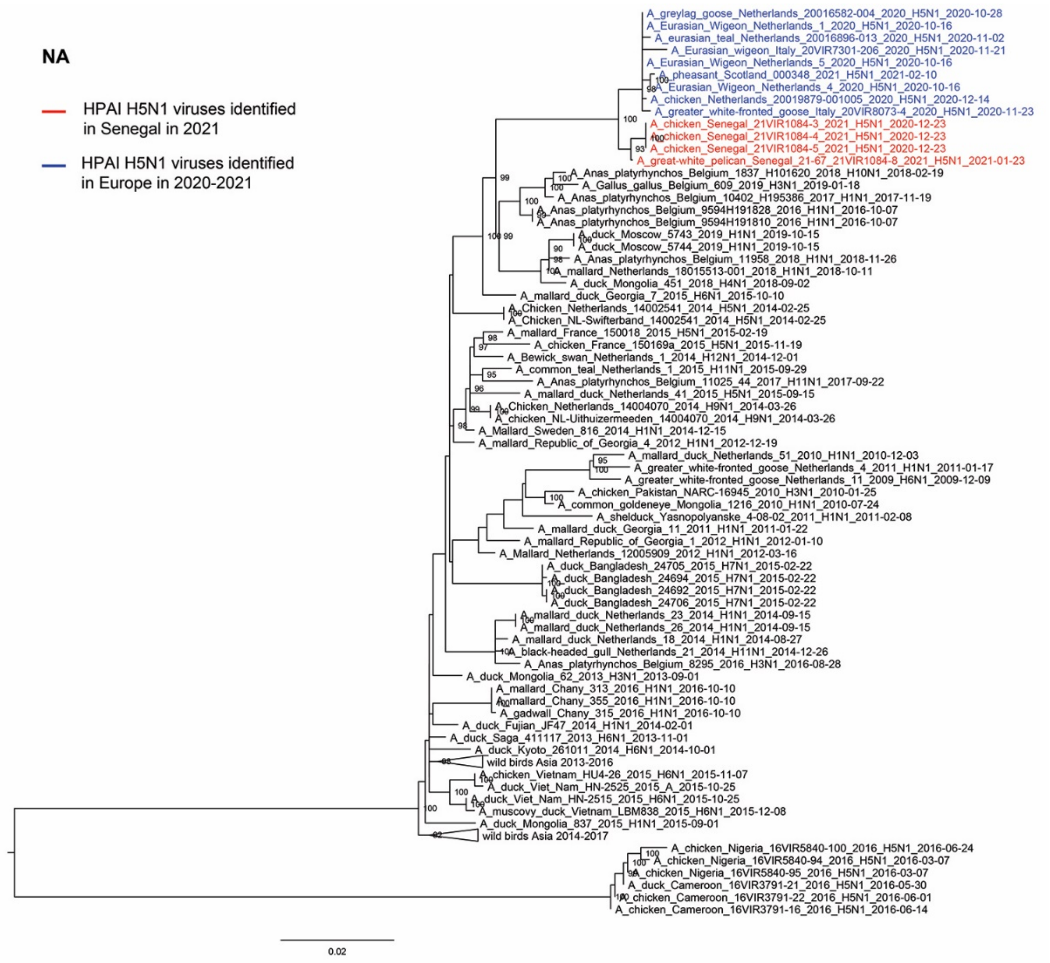

Appendix Figure 2. Maximum likelihood phylogenetic tree of the neuraminidase (NA) gene obtained by using IQTREE version 1.6.6. The HPAI H5N1 viruses from Senegal are marked in red; the HPAI H5N1 viruses from Europe are marked in blue. Ultrafast bootstrap supports >80 are indicated next to the nodes. HPAI, highly pathogenic avian influenza.

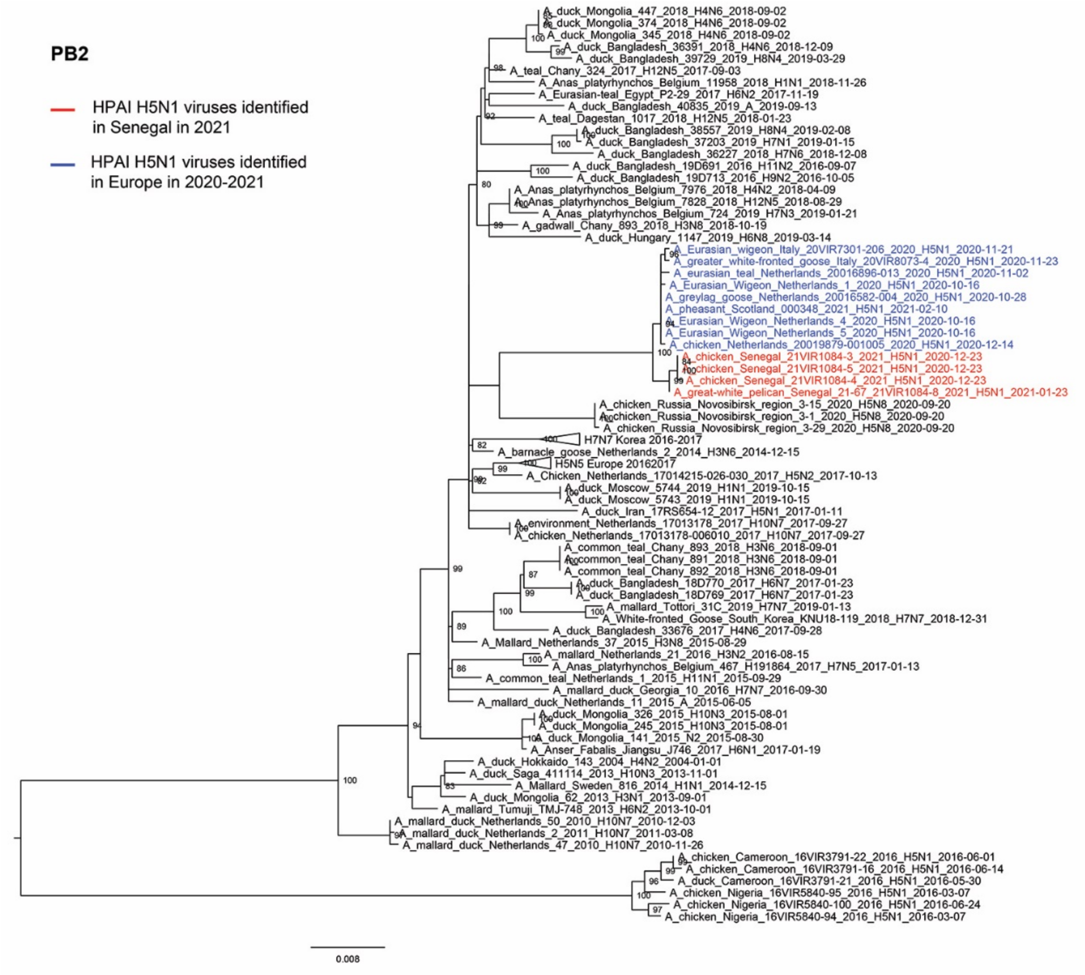

Appendix Figure 3. Maximum likelihood phylogenetic tree of the polymerase basic 2 (PB2) gene obtained by using IQTREE version 1.6.6. The HPAI H5N1 viruses from Senegal are marked in red; the HPAI H5N1 viruses from Europe are marked in blue. Ultrafast bootstrap supports >80 are indicated next to the nodes. HPAI, highly pathogenic avian influenza.

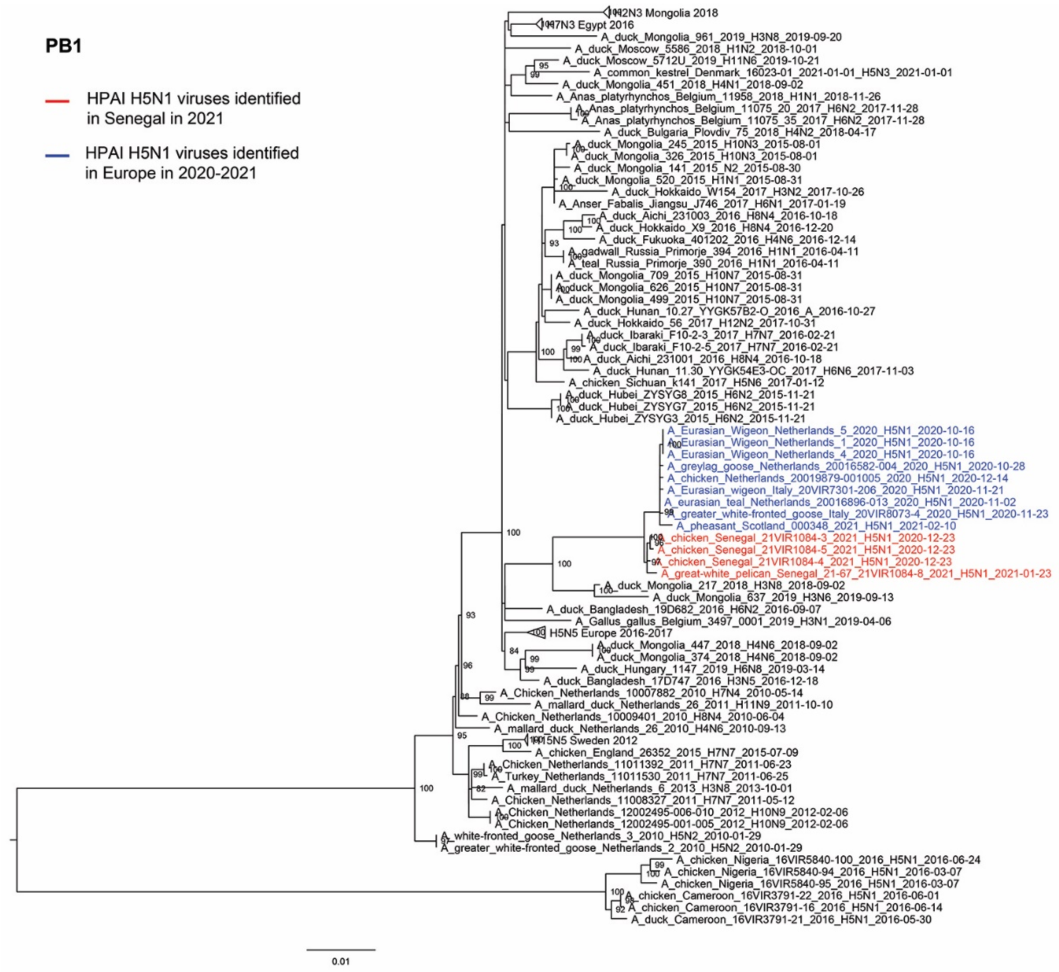

Appendix Figure 4. Maximum likelihood phylogenetic tree of the polymerase basic 1 (PB1) gene obtained by using IQTREE version 1.6.6. The HPAI H5N1 viruses from Senegal are marked in red; the HPAI H5N1 viruses from Europe are marked in blue. Ultrafast bootstrap supports >80 are indicated next to the nodes. HPAI, highly pathogenic avian influenza.

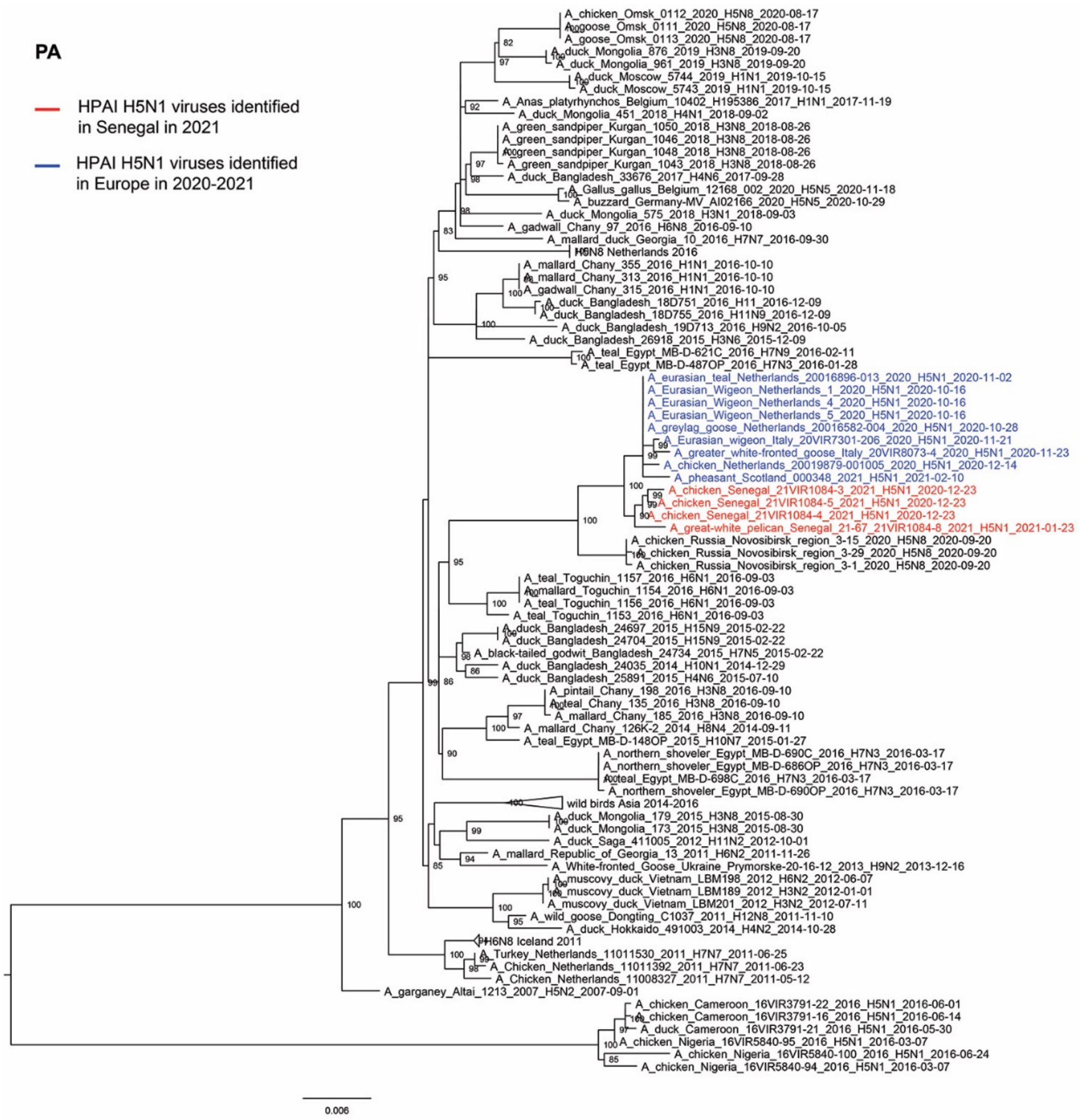

Appendix Figure 5. Maximum likelihood phylogenetic tree of the polymerase acidic (PA) gene obtained by using IQTREE version 1.6.6. The HPAI H5N1 viruses from Senegal are marked in red; the HPAI H5N1 viruses from Europe are marked in blue. Ultrafast bootstrap supports >80 are indicated next to the nodes. HPAI, highly pathogenic avian influenza.

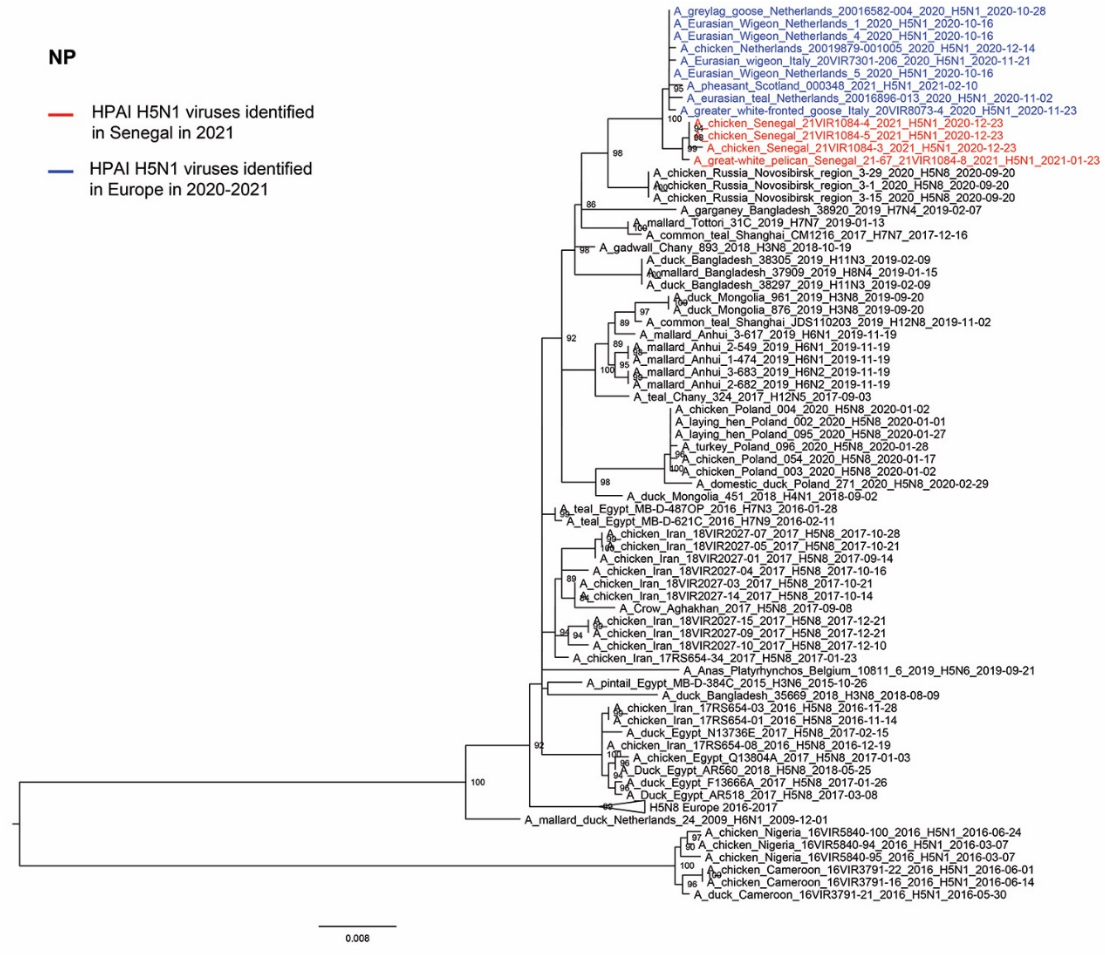

Appendix Figure 6. Maximum likelihood phylogenetic tree of the nucleoprotein (NP) gene obtained by using IQTREE version 1.6.6. The HPAI H5N1 viruses from Senegal are marked in red; the HPAI H5N1 viruses from Europe are marked in blue. Ultrafast bootstrap supports >80 are indicated next to the nodes. HPAI, highly pathogenic avian influenza.

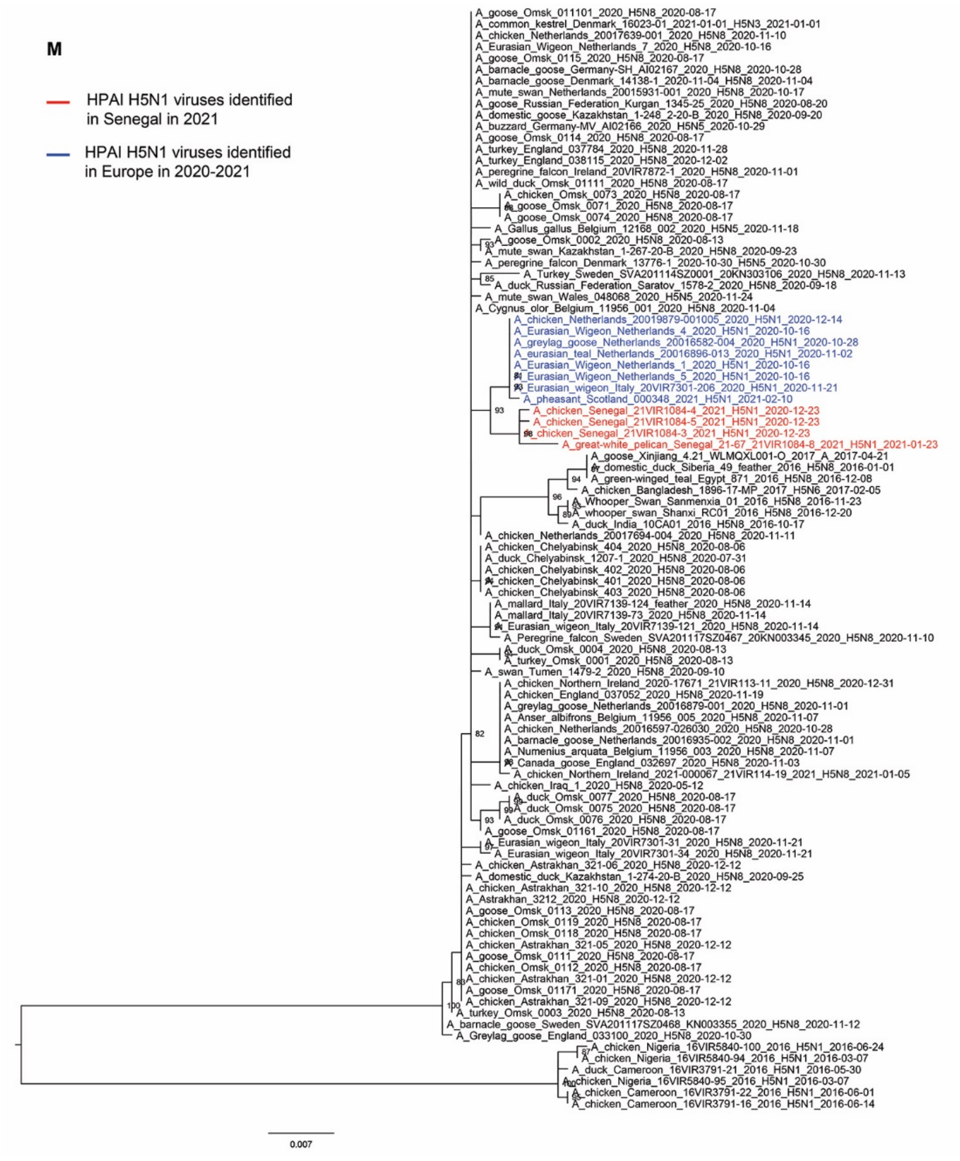

Appendix Figure 7. Maximum likelihood phylogenetic tree of the matrix (M) gene obtained by using IQTREE version 1.6.6. The HPAI H5N1 viruses from Senegal are marked in red; the HPAI H5N1 viruses from Europe are marked in blue. Ultrafast bootstrap supports >80 are indicated next to the nodes. HPAI, highly pathogenic avian influenza.

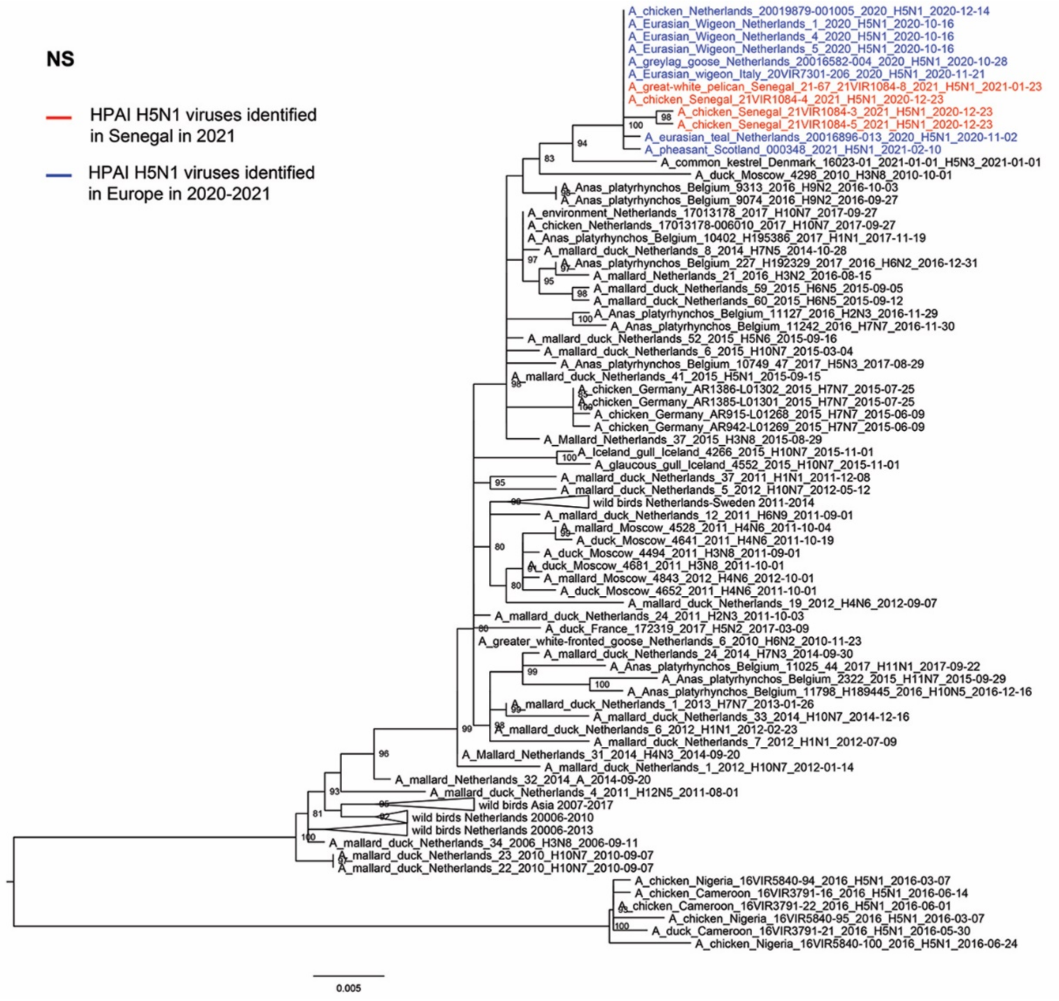

Appendix Figure 8. Maximum likelihood phylogenetic tree of the non-structural (NS) gene obtained by using IQTREE version 1.6.6. The HPAI H5N1 viruses from Senegal are marked in red; the HPAI H5N1 viruses from Europe are marked in blue. Ultrafast bootstrap supports >80 are indicated next to the nodes. HPAI, highly pathogenic avian influenza.

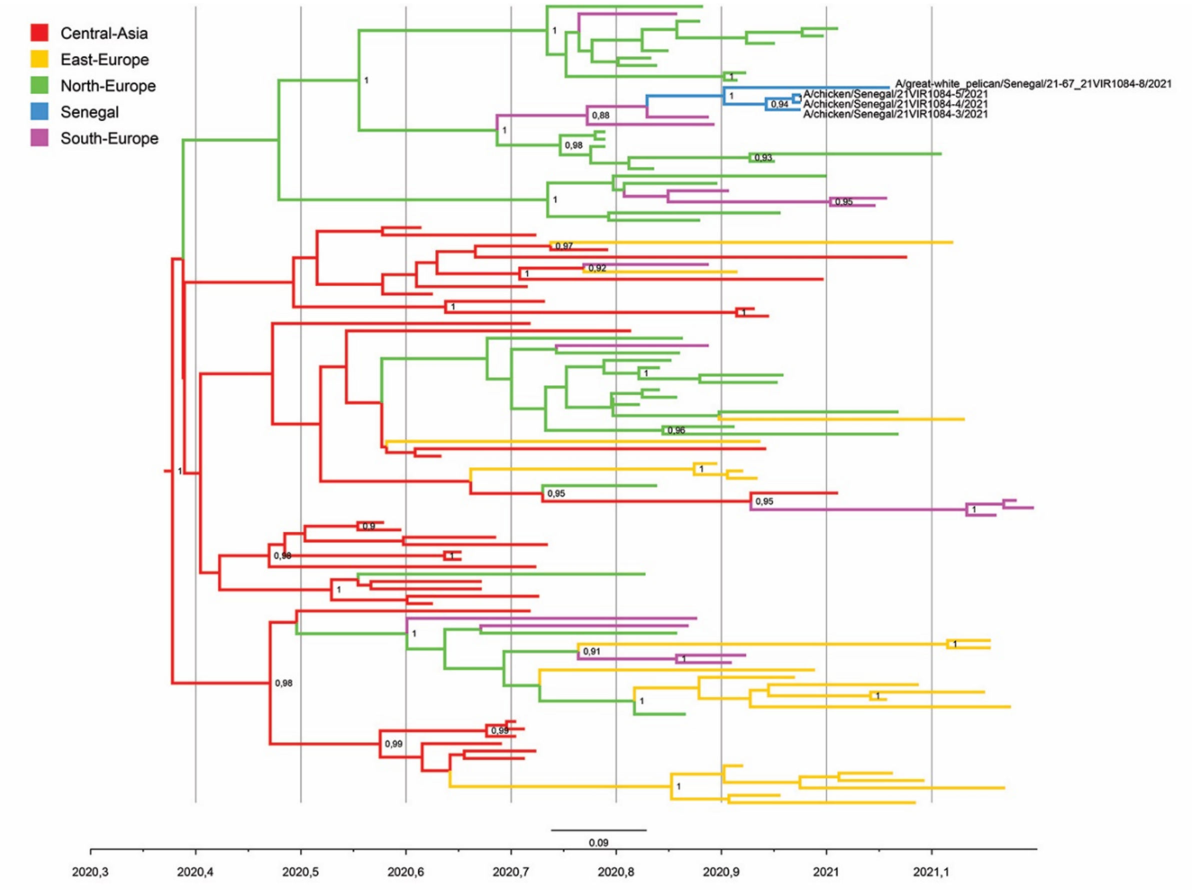
Central-Asia
East-Europe
North-Europe
Senegal
South-Europe
A/great-white_pelican/Senegal/21-67_21VIR1084-8/2021
A/chicken/Senegal/21VIR1084-5/2021
A/chicken/Senegal/21VIR1084-4/2021
A/chicken/Senegal/21VIR1084-3/2021
0.09
2020,3
2020,4
2020,5
2020,6
2020,7
2020,8
2020,9
2021
2021,1

Appendix Table. Acknowledgment table of the authors and the originating and submitting laboratories of the sequences from the GISAID EpiFlu Database on which this research is partly based

Segment ID	Country	Collection date	Isolate-ID	Isolate name	Originating Laboratory	Submitting Laboratory	Authors
EPI1813749	Denmark	2020 Oct 30	EPI_ISL_644737	A/peregrine_falcon/Denmark/13776–1/2020–10–30	Statens Serum Institut	Statens Serum Institut	Charlotte Hjulsager
EPI1814727	Sweden	2020 Nov 10	EPI_ISL_668456	A/Peregrine_falcon/Sweden/SVA201117SZ0467/20KN003345/2020	National Veterinary Institute, SVA	National Veterinary Institute	
EPI1847780	Denmark	2021 Jan 1	EPI_ISL_1063993	A/common_kestrel/Denmark/16023–01/2021–01–01	Statens Serum Institut	Statens Serum Institut	Charlotte Hjulsager, Jesper Schak Krog
EPI1843642	Italy	2020 Nov 23	EPI_ISL_956412	A/greater_white-fronted_goose/Italy/20VIR8073–4/2020	Istituto Zooprofilattico Sperimentale delle Venezie, EU/OIE/Reference Laboratory and FAO Reference Centre for AI and ND	Istituto Zooprofilattico Sperimentale Delle Venezie	B. Zecchin, A. Fusaro, A. Milani, A. Schivo, A. Salviato, A. Pastori, G. Zamperin, I. Monne, C. Terregino
EPI1811671	Russian Federation	2020 Oct 2	EPI_ISL_626648	A/goose/Russian_Federation/Omsk/1680–6/2020	Federal Centre for Animal Health (ARRIAH)	Animal and Plant Health Agency (APHA)	
EPI1814684	Russian Federation	2020 Sep 10	EPI_ISL_661178	A/swan/Tumen/1479–2/2020	Federal Centre for Animal Health (ARRIAH) OIE Regional Reference Laboratory	Federal Centre for Animal Health (ARRIAH)	N. Zinyakov, P. Akshalova, P. Zhestkov, A. Kozlov, A. Andriyasov, E. Ovchinnikova, Z. Nikonova, V. Sosipatorova, L. Scherbakova, D. Andreychuk, I. Chvala
EPI1811584	Kazakhstan	2020 Sep 23	EPI_ISL_614401	A/mute_swan/Kazakhstan/1–267–20-B/2020	National Veterinary Reference Center	Animal and Plant Health Agency (APHA)	Elliot Whittard
EPI1813730	Belgium	2020 Nov 4	EPI_ISL_644735	A/Cygnus_olor/Belgium/11956_001/2020	Sciensano - Animal Infectious Diseases	Sciensano, Department of Animal Infectious Diseases	Steven Van Borm, Elisabeth Mathijs, Thierry van den Berg, Bénédicte Lambrecht, Mieke Steensels
EPI1815183	United Kingdom	2020 Nov 24	EPI_ISL_683999	A/mute_swan/Wales/048068/2020	Animal and Plant Health Agency (APHA)	Animal and Plant Health Agency (APHA)	
EPI1806859	Netherlands	2020 Oct 17	EPI_ISL_591075	A/mute_swan/Netherlands/20015931–001/2020	Wageningen Bioveterinary Research	Wageningen Bioveterinary Research	Nancy Beerens, Frank Harders, Sylvia Verschuren-Pritz, Marit Roose, Evelien Germeraad, Marc Engelsma, Alex Bossers, Rene Heutink
EPI1837949	United Kingdom	2020 Dec 2	EPI_ISL_710505	A/turkey/England/038115/2020	Animal and Plant Health Agency (APHA)	Animal and Plant Health Agency (APHA)	
EPI1837933	United Kingdom	2020 Nov 28	EPI_ISL_710504	A/turkey/England/037784/2020	Animal and Plant Health Agency (APHA)	Animal and Plant Health Agency (APHA)	
EPI1846961	Russian Federation	2020 Dec 12	EPI_ISL_1038924	A/Astrakhan/3212/2020	Center of Hygiene and Epidemiology in Astrakhan Region	State Research Center of Virology and Biotechnology (VECTOR)	O. Pyankova, I. Susloparov, V. Marchenko, A. Ryzhikov

Segment ID	Country	Collection date	Isolate-ID	Isolate name	Originating Laboratory	Submitting Laboratory	Authors
EPI1807267	Netherlands	2020 Oct 16	EPI_ISL_603136	A/Eurasian_Wigeon/Netherlands/7/2020	Erasmus Medical Center	Erasmus Medical Center	
EPI1807259	Netherlands	2020 Oct 16	EPI_ISL_603135	A/Eurasian_Wigeon/Netherlands/5/2020	Erasmus Medical Center	Erasmus Medical Center	
EPI1807251	Netherlands	2020 Oct 16	EPI_ISL_603134	A/Eurasian_Wigeon/Netherlands/4/2020	Erasmus Medical Center	Erasmus Medical Center	
EPI1807243	Netherlands	2020 Oct 16	EPI_ISL_603133	A/Eurasian_Wigeon/Netherlands/1/2020	Erasmus Medical Center	Erasmus Medical Center	
EPI1846305	United Kingdom	2021 Jan 5	EPI_ISL_996003	A/chicken/Northern_Ireland/2021–000067_21VIR114–19/2021	AFBI - Agri-Food & Bioscience Institute	Istituto Zooprofilattico Sperimentale Delle Venezie	M.J. McMenamy, V. Harkin, K. Lemon, B. Zecchin, A. Fusaro, A. Schivo, A. Salviato, A. Pastori, I. Monne, C. Terregino
EPI1846297	United Kingdom	2020 Dec 31	EPI_ISL_995172	A/chicken/Northern_Ireland/2020–17671_21VIR113–11/2020	AFBI - Agri-Food & Bioscience Institute	Istituto Zooprofilattico Sperimentale Delle Venezie	M.J. McMenamy, V. Harkin, K. Lemon, B. Zecchin, A. Fusaro, A. Schivo, A. Salviato, A. Pastori, I. Monne, C. Terregino
EPI1811657	Russian Federation	2020 Oct 2	EPI_ISL_626647	A/chicken/Russian_Federation/Omsk/1680–10/2020	Federal Centre for Animal Health (ARRIAH)	Animal and Plant Health Agency (APHA)	
EPI1811628	Iraq	2020 May 12	EPI_ISL_623074	A/chicken/Iraq/1/2020	Central Veterinary Labs	Animal and Plant Health Agency (APHA)	
EPI1838673	Netherlands	2020 Dec 14	EPI_ISL_711055	A/chicken/Netherlands/20019879–001005/2020	Wageningen Bioveterinary Research	Wageningen Bioveterinary Research	Nancy Beerens, Frank Harders, Sylvia Verschuren-Pritz, Marit Roose, Evelien Germeraad, Marc Engelsma, Alex Bossers, Rene Heutink
EPI1813085	Netherlands	2020 Nov 11	EPI_ISL_641395	A/chicken/Netherlands/20017694–004/2020	Wageningen Bioveterinary Research	Wageningen Bioveterinary Research	Nancy Beerens, Frank Harders, Sylvia Verschuren-Pritz, Marit Roose, Evelien Germeraad, Marc Engelsma, Alex Bossers, Rene Heutink
EPI1813077	Netherlands	2020 Nov 10	EPI_ISL_641394	A/chicken/Netherlands/20017639–001/2020	Wageningen Bioveterinary Research	Wageningen Bioveterinary Research	Nancy Beerens, Frank Harders, Sylvia Verschuren-Pritz, Marit Roose, Evelien Germeraad, Marc Engelsma, Alex Bossers, Rene Heutink
EPI1812965	Netherlands	2020 Nov 2	EPI_ISL_641377	A/chicken/Netherlands/20016978–001/2020	Wageningen Bioveterinary Research	Wageningen Bioveterinary Research	Nancy Beerens, Frank Harders, Sylvia Verschuren-Pritz, Marit Roose, Evelien Germeraad, Marc Engelsma, Alex Bossers, Rene Heutink

Segment ID	Country	Collection date	Isolate-ID	Isolate name	Originating Laboratory	Submitting Laboratory	Authors
EPI1812533	Russian Federation	2020 Jul 31	EPI_ISL_637098	A/duck/Chelyabinsk/1207–1/2020	Federal Centre for Animal Health (ARRIAH) OIE Regional Reference Laboratory	Federal Centre for Animal Health (ARRIAH)	N. Zinyakov, P. Akshalova, P. Zhestkov, A. Kozlov, A. Andriyasov, E. Ovchinnikova, Z. Nikonova, V. Sosipatorova, L. Scherbakova, D. Andreychuk, I. Chvala
EPI1837899	United Kingdom	2020 Nov 18	EPI_ISL_710512	A/whistling_duck/England/035643/2020	Animal and Plant Health Agency (APHA)	Animal and Plant Health Agency (APHA)	
EPI1811696	Russian Federation	2020 Aug 17	EPI_ISL_626650	A/duck/Russian_Federation/Omsk/1328–2/2020	Federal Centre for Animal Health (ARRIAH)	Animal and Plant Health Agency (APHA)	
EPI1811611	Kazakhstan	2020 Sep 25	EPI_ISL_615072	A/domestic_duck/Kazakhstan/1–274–20-B/2020	National Veterinary Reference Center	Animal and Plant Health Agency (APHA)	
EPI1813249	Russian Federation	2020 Aug 17	EPI_ISL_644138	A/wild_duck/Omsk/01111/2020	State Research Center of Virology and Biotechnology (VECTOR)	State Research Center of Virology and Biotechnology (VECTOR)	Natalia Goncharova, Ivan Susloparov, Natalia Kolosova, Alexey Danilenko, Juliya Bulanovich, Vasiliy Marchenko, Alexander Ryzhikov
EPI1811564	Germany	2020 Oct 29	EPI_ISL_614399	A/buzzard/Germany-MV/AI02166/2020	Landesamt für Landwirtschaft, Lebensmittelsicherheit und Fischerei (LALLF)	Friedrich-Loeffler-Institut	
EPI1837941	United Kingdom	2020 Nov 3	EPI_ISL_710506	A/Canada_goose/England/032697/2020	Animal and Plant Health Agency (APHA)	Animal and Plant Health Agency (APHA)	
EPI1841769	Ireland	2020 Nov 1	EPI_ISL_813979	A/peregrine_falcon/Ireland/20VIR7872–1/2020	Central Veterinary Research Laboratory	Istituto Zooprofilattico Sperimentale Delle Venezie	O. Flynn, E. Connaghan, C. Byrne, L. Garza Cuartero, R. O´Neill, B. Zecchin, A. Fusaro, A. Pastori, A. Salviato, A. Schivo, I. Monne, C. Terregino
EPI1815373	Italy	2020 Nov 21	EPI_ISL_683752	A/Eurasian_wigeon/Italy/20VIR7301–34/2020	Istituto Zooprofilattico Sperimentale delle Venezie, EU/OIE/Reference Laboratory and FAO Reference Centre for AI and ND	Istituto Zooprofilattico Sperimentale Delle Venezie	
EPI1815381	Italy	2020 Nov 21	EPI_ISL_683751	A/Eurasian_wigeon/Italy/20VIR7301–31/2020	Istituto Zooprofilattico Sperimentale delle Venezie, EU/OIE/Reference Laboratory and FAO Reference Centre for AI and ND	Istituto Zooprofilattico Sperimentale Delle Venezie	

Segment ID	Country	Collection date	Isolate-ID	Isolate name	Originating Laboratory	Submitting Laboratory	Authors
EPI1815150	Italy	2020 Nov 14	EPI_ISL_683593	A/Eurasian_wigeon/Italy/20VIR7139–121/2020	Istituto Zooprofilattico Sperimentale delle Venezie, EU/OIE/Reference Laboratory and FAO Reference Centre for AI and ND	Istituto Zooprofilattico Sperimentale Delle Venezie	B. Zecchin, A. Fusaro, A. Pastori, A. Milani, A. Salviato, A. Schivo, I. Monne, C. Terregino
EPI1815142	Italy	2020 Nov 21	EPI_ISL_683592	A/Eurasian_wigeon/Italy/20VIR7301–206/2020	Istituto Zooprofilattico Sperimentale delle Venezie, EU/OIE/Reference Laboratory and FAO Reference Centre for AI and ND	Istituto Zooprofilattico Sperimentale Delle Venezie	B. Zecchin, A. Fusaro, A. Pastori, A. Milani, A. Salviato, A. Schivo, I. Monne, C. Terregino
EPI1815159	Italy	2020 Nov 14	EPI_ISL_683594	A/mallard/Italy/20VIR7139–124_feather/2020	Istituto Zooprofilattico Sperimentale delle Venezie, EU/OIE/Reference Laboratory and FAO Reference Centre for AI and ND	Istituto Zooprofilattico Sperimentale Delle Venezie	B. Zecchin, A. Fusaro, A. Pastori, A. Milani, A. Salviato, A. Schivo, I. Monne, C. Terregino
EPI1814606	Italy	2020 Nov 14	EPI_ISL_654958	A/mallard/Italy/20VIR7139–73/2020	Istituto Zooprofilattico Sperimentale delle Venezie, EU/OIE/Reference Laboratory and FAO Reference Centre for AI and ND	Istituto Zooprofilattico Sperimentale Delle Venezie	B. Zecchin, A. Fusaro, A. Pastori, A. Milani, A. Salviato, A. Schivo, I. Monne, C. Terregino
EPI1814694	Belgium	2020 Nov 7	EPI_ISL_664102	A/Numenius_arquata/Belgium/11956_003/2020	Sciensano - Animal Infectious Diseases	Sciensano, Department of Animal Infectious Diseases	Steven Van Borm, Elisabeth Mathijs, Frank Vandenbussche, Thierry van den Berg, Bénédicte Lambrecht, Mieke Steensels
EPI1812399	Netherlands	2020 Nov 1	EPI_ISL_632318	A/greylag_goose/Netherlands/20016879–001/2020	Wageningen Bioveterinary Research	Wageningen Bioveterinary Research	Nancy Beerens, Frank Harders, Sylvia Verschuren-Pritz, Marit Roose, Evelien Germeraad, Marc Engelsma, Alex Bossers, Rene Heutink
EPI1812367	Netherlands	2020 Oct 28	EPI_ISL_632314	A/greylag_goose/Netherlands/20016582–004/2020	Wageningen Bioveterinary Research	Wageningen Bioveterinary Research	Nancy Beerens, Frank Harders, Sylvia Verschuren-Pritz, Marit Roose, Evelien Germeraad, Marc Engelsma, Alex Bossers, Rene Heutink
EPI1813753	Denmark	2020 Nov 4	EPI_ISL_644824	A/barnacle_goose/Denmark/14138–1/2020–11–04	Statens Serum Institut	Statens Serum Institut	Charlotte,Hjulsager

Segment ID	Country	Collection date	Isolate-ID	Isolate name	Originating Laboratory	Submitting Laboratory	Authors
EPI1811572	Germany	2020 Oct 28	EPI_ISL_614400	A/barnacle_goose/Germany-SH/AI02167/2020	Landeslabor Schleswig-Holstein	Friedrich-Loeffler-Institut	
EPI1812391	Netherlands	2020 Nov 1	EPI_ISL_632317	A/barnacle_goose/Netherlands/20016935–002/2020	Wageningen Bioveterinary Research	Wageningen Bioveterinary Research	Nancy Beerens, Frank Harders, Sylvia Verschuren-Pritz, Marit Roose, Evelien Germeraad, Marc Engelsma, Alex Bossers, Rene Heutink
EPI1814735	Sweden	2020 Nov 12	EPI_ISL_668457	A/barnacle_goose/Sweden/SVA201117SZ0468/KN003355/2020	National Veterinary Institute, SVA	National Veterinary Institute	
EPI1814627	Belgium	2020 Nov 18	EPI_ISL_660264	A/Gallus_gallus/Belgium/12168_002/2020	Sciensano - Animal Infectious Diseases	Sciensano, Department of Animal Infectious Diseases	Steven Van Borm, Elisabeth Mathijs, Frank Vandenbussche, Thierry van den Berg, Bénédicte Lambrecht, Mieke Steensels
EPI1844083	Czech Republic	2021 Jan 22	EPI_ISL_977513	A/chicken/Czech_Republic/1566–1/2021	State Veterinary Institute Prague	State Veterinary Institute Prague	A. Nagy, L. Cernikova, M. Stara
EPI1847025	Russian Federation	2020 Dec 12	EPI_ISL_1039240	A/chicken/Astrakhan/321–10/2020	State Research Center of Virology and Biotechnology (VECTOR)	State Research Center of Virology and Biotechnology (VECTOR)	Natalia Goncharova, Ivan Susloparov, Natalia Kolosova, Alexey Danilenko, Juliya Bulanovich, Vasiliy Marchenko, Alexander Ryzhikov
EPI1847017	Russian Federation	2020 Dec 12	EPI_ISL_1039239	A/chicken/Astrakhan/321–10/2020	State Research Center of Virology and Biotechnology (VECTOR)	State Research Center of Virology and Biotechnology (VECTOR)	Natalia Goncharova, Ivan Susloparov, Natalia Kolosova, Alexey Danilenko, Juliya Bulanovich, Vasiliy Marchenko, Alexander Ryzhikov
EPI1847009	Russian Federation	2020 Dec 12	EPI_ISL_1039238	A/chicken/Astrakhan/321–09/2020	State Research Center of Virology and Biotechnology (VECTOR)	State Research Center of Virology and Biotechnology (VECTOR)	Natalia Goncharova, Ivan Susloparov, Natalia Kolosova, Alexey Danilenko, Juliya Bulanovich, Vasiliy Marchenko, Alexander Ryzhikov
EPI1847001	Russian Federation	2020 Dec 12	EPI_ISL_1039236	A/chicken/Astrakhan/321–06/2020	State Research Center of Virology and Biotechnology (VECTOR)	State Research Center of Virology and Biotechnology (VECTOR)	Natalia Goncharova, Ivan Susloparov, Natalia Kolosova, Alexey Danilenko, Juliya Bulanovich, Vasiliy Marchenko, Alexander Ryzhikov

Segment ID	Country	Collection date	Isolate-ID	Isolate name	Originating Laboratory	Submitting Laboratory	Authors
EPI1846993	Russian Federation	2020 Dec 12	EPI_ISL_1039235	A/chicken/Astrakhan/321–05/2020	State Research Center of Virology and Biotechnology (VECTOR)	State Research Center of Virology and Biotechnology (VECTOR)	Natalia Goncharova, Ivan Susloparov, Natalia Kolosova, Alexey Danilenko, Juliya Bulanovich, Vasiliy Marchenko, Alexander Ryzhikov
EPI1846985	Russian Federation	2020 Dec 12	EPI_ISL_1039234	A/chicken/Astrakhan/321–05/2020	State Research Center of Virology and Biotechnology (VECTOR)	State Research Center of Virology and Biotechnology (VECTOR)	
EPI1846977	Russian Federation	2020 Dec 12	EPI_ISL_1039232	A/chicken/Astrakhan/321–01/2020	State Research Center of Virology and Biotechnology (VECTOR)	State Research Center of Virology and Biotechnology (VECTOR)	
EPI1846969	Russian Federation	2020 Dec 12	EPI_ISL_1039231	A/chicken/Astrakhan/321–01/2020	State Research Center of Virology and Biotechnology (VECTOR)	State Research Center of Virology and Biotechnology (VECTOR)	
EPI1813433	Russian Federation	2020 Aug 6	EPI_ISL_644161	A/chicken/Chelyabinsk/404/2020	State Research Center of Virology and Biotechnology (VECTOR)	State Research Center of Virology and Biotechnology (VECTOR)	Natalia Goncharova, Ivan Susloparov, Natalia Kolosova, Alexey Danilenko, Juliya Bulanovich, Vasiliy Marchenko, Alexander Ryzhikov
EPI1813425	Russian Federation	2020 Aug 6	EPI_ISL_644160	A/chicken/Chelyabinsk/403/2020	State Research Center of Virology and Biotechnology (VECTOR)	State Research Center of Virology and Biotechnology (VECTOR)	Natalia Goncharova, Ivan Susloparov, Natalia Kolosova, Alexey Danilenko, Juliya Bulanovich, Vasiliy Marchenko, Alexander Ryzhikov
EPI1813409	Russian Federation	2020 Aug 17	EPI_ISL_644158	A/chicken/Omsk/0073/2020	State Research Center of Virology and Biotechnology (VECTOR)	State Research Center of Virology and Biotechnology (VECTOR)	Natalia Goncharova, Ivan Susloparov, Natalia Kolosova, Alexey Danilenko, Juliya Bulanovich, Vasiliy Marchenko, Alexander Ryzhikov
EPI1813385	Russian Federation	2020 Aug 17	EPI_ISL_644155	A/chicken/Omsk/0119/2020	State Research Center of Virology and Biotechnology (VECTOR)	State Research Center of Virology and Biotechnology (VECTOR)	Natalia Goncharova, Ivan Susloparov, Natalia Kolosova, Alexey Danilenko, Juliya Bulanovich, Vasiliy Marchenko, Alexander Ryzhikov

Segment ID	Country	Collection date	Isolate-ID	Isolate name	Originating Laboratory	Submitting Laboratory	Authors
EPI1813377	Russian Federation	2020 Aug 17	EPI_ISL_644154	A/chicken/Omsk/0118/2020	State Research Center of Virology and Biotechnology (VECTOR)	State Research Center of Virology and Biotechnology (VECTOR)	Natalia Goncharova, Ivan Susloparov, Natalia Kolosova, Alexey Danilenko, Juliya Bulanovich, Vasiliy Marchenko, Alexander Ryzhikov
EPI1813345	Russian Federation	2020 Aug 17	EPI_ISL_644150	A/chicken/Omsk/0112/2020	State Research Center of Virology and Biotechnology (VECTOR)	State Research Center of Virology and Biotechnology (VECTOR)	Natalia Goncharova, Ivan Susloparov, Natalia Kolosova, Alexey Danilenko, Juliya Bulanovich, Vasiliy Marchenko, Alexander Ryzhikov
EPI1813329	Russian Federation	2020 Aug 6	EPI_ISL_644148	A/chicken/Chelyabinsk/404/2020	State Research Center of Virology and Biotechnology (VECTOR)	State Research Center of Virology and Biotechnology (VECTOR)	Natalia Goncharova, Ivan Susloparov, Natalia Kolosova, Alexey Danilenko, Juliya Bulanovich, Vasiliy Marchenko, Alexander Ryzhikov
EPI1813321	Russian Federation	2020 Aug 6	EPI_ISL_644147	A/chicken/Chelyabinsk/403/2020	State Research Center of Virology and Biotechnology (VECTOR)	State Research Center of Virology and Biotechnology (VECTOR)	Natalia Goncharova, Ivan Susloparov, Natalia Kolosova, Alexey Danilenko, Juliya Bulanovich, Vasiliy Marchenko, Alexander Ryzhikov
EPI1813313	Russian Federation	2020 Aug 6	EPI_ISL_644146	A/chicken/Chelyabinsk/402/2020	State Research Center of Virology and Biotechnology (VECTOR)	State Research Center of Virology and Biotechnology (VECTOR)	Natalia Goncharova, Ivan Susloparov, Natalia Kolosova, Alexey Danilenko, Juliya Bulanovich, Vasiliy Marchenko, Alexander Ryzhikov
EPI1813305	Russian Federation	2020 Aug 6	EPI_ISL_644145	A/chicken/Chelyabinsk/401/2020	State Research Center of Virology and Biotechnology (VECTOR)	State Research Center of Virology and Biotechnology (VECTOR)	Natalia Goncharova, Ivan Susloparov, Natalia Kolosova, Alexey Danilenko, Juliya Bulanovich, Vasiliy Marchenko, Alexander Ryzhikov
EPI1813265	Russian Federation	2020 Aug 17	EPI_ISL_644140	A/chicken/Omsk/0073/2020	State Research Center of Virology and Biotechnology (VECTOR)	State Research Center of Virology and Biotechnology (VECTOR)	Natalia Goncharova, Ivan Susloparov, Natalia Kolosova, Alexey Danilenko, Juliya Bulanovich, Vasiliy

Segment ID	Country	Collection date	Isolate-ID	Isolate name	Originating Laboratory	Submitting Laboratory	Authors
							Marchenko, Alexander Ryzhikov
EPI1813225	Russian Federation	2020 Aug 17	EPI_ISL_644135	A/chicken/Omsk/0119/2020	State Research Center of Virology and Biotechnology (VECTOR)	State Research Center of Virology and Biotechnology (VECTOR)	Natalia Goncharova, Ivan Susloparov, Natalia Kolosova, Alexey Danilenko, Juliya Bulanovich, Vasiliy Marchenko, Alexander Ryzhikov
EPI1813217	Russian Federation	2020 Aug 17	EPI_ISL_644134	A/chicken/Omsk/0118/2020	State Research Center of Virology and Biotechnology (VECTOR)	State Research Center of Virology and Biotechnology (VECTOR)	Natalia Goncharova, Ivan Susloparov, Natalia Kolosova, Alexey Danilenko, Juliya Bulanovich, Vasiliy Marchenko, Alexander Ryzhikov
EPI1813153	Russian Federation	2020 Aug 17	EPI_ISL_644126	A/chicken/Omsk/0112/2020	State Research Center of Virology and Biotechnology (VECTOR)	State Research Center of Virology and Biotechnology (VECTOR)	Natalia Goncharova, Ivan Susloparov, Natalia Kolosova, Alexey Danilenko, Juliya Bulanovich, Vasiliy Marchenko, Alexander Ryzhikov
EPI1837917	United Kingdom	2020 Nov 9	EPI_ISL_710509	A/chicken/England/033708/2020	Animal and Plant Health Agency (APHA)	Animal and Plant Health Agency (APHA)	
EPI1807231	Netherlands	2020 Oct 28	EPI_ISL_603132	A/chicken/Netherlands/20016597–026030/2020	Wageningen Bioveterinary Research	Wageningen Bioveterinary Research	Nancy Beerens, Frank Harders, Sylvia Verschuren-Pritz, Marit Roose, Evelien Germeraad, Marc Engelsma, Alex Bossers, Rene Heutink
EPI1813393	Russian Federation	2020 Aug 17	EPI_ISL_644156	A/goose/Omsk/011101/2020	State Research Center of Virology and Biotechnology (VECTOR)	State Research Center of Virology and Biotechnology (VECTOR)	Natalia Goncharova, Ivan Susloparov, Natalia Kolosova, Alexey Danilenko, Juliya Bulanovich, Vasiliy Marchenko, Alexander Ryzhikov
EPI1813361	Russian Federation	2020 Aug 17	EPI_ISL_644152	A/goose/Omsk/01161/2020	State Research Center of Virology and Biotechnology (VECTOR)	State Research Center of Virology and Biotechnology (VECTOR)	Natalia Goncharova, Ivan Susloparov, Natalia Kolosova, Alexey Danilenko, Juliya Bulanovich, Vasiliy Marchenko, Alexander Ryzhikov
EPI1813353	Russian Federation	2020 Aug 17	EPI_ISL_644151	A/goose/Omsk/0114/2020	State Research Center of Virology and	State Research Center of Virology and Biotechnology (VECTOR)	Natalia Goncharova, Ivan Susloparov, Natalia Kolosova, Alexey

Segment ID	Country	Collection date	Isolate-ID	Isolate name	Originating Laboratory	Submitting Laboratory	Authors
					Biotechnology (VECTOR)		Danilenko, Juliya Bulanovich, Vasiliy Marchenko, Alexander Ryzhikov
EPI1813273	Russian Federation	2020 Aug 17	EPI_ISL_644141	A/goose/Omsk/0074/2020	State Research Center of Virology and Biotechnology (VECTOR)	State Research Center of Virology and Biotechnology (VECTOR)	Natalia Goncharova, Ivan Susloparov, Natalia Kolosova, Alexey Danilenko, Juliya Bulanovich, Vasiliy Marchenko, Alexander Ryzhikov
EPI1813257	Russian Federation	2020 Aug 17	EPI_ISL_644139	A/goose/Omsk/0071/2020	State Research Center of Virology and Biotechnology (VECTOR)	State Research Center of Virology and Biotechnology (VECTOR)	Natalia Goncharova, Ivan Susloparov, Natalia Kolosova, Alexey Danilenko, Juliya Bulanovich, Vasiliy Marchenko, Alexander Ryzhikov
EPI1813233	Russian Federation	2020 Aug 17	EPI_ISL_644136	A/goose/Omsk/011101/2020	State Research Center of Virology and Biotechnology (VECTOR)	State Research Center of Virology and Biotechnology (VECTOR)	Natalia Goncharova, Ivan Susloparov, Natalia Kolosova, Alexey Danilenko, Juliya Bulanovich, Vasiliy Marchenko, Alexander Ryzhikov
EPI1813201	Russian Federation	2020 Aug 17	EPI_ISL_644132	A/goose/Omsk/01171/2020	State Research Center of Virology and Biotechnology (VECTOR)	State Research Center of Virology and Biotechnology (VECTOR)	Natalia Goncharova, Ivan Susloparov, Natalia Kolosova, Alexey Danilenko, Juliya Bulanovich, Vasiliy Marchenko, Alexander Ryzhikov
EPI1813185	Russian Federation	2020 Aug 17	EPI_ISL_644130	A/goose/Omsk/01161/2020	State Research Center of Virology and Biotechnology (VECTOR)	State Research Center of Virology and Biotechnology (VECTOR)	Natalia Goncharova, Ivan Susloparov, Natalia Kolosova, Alexey Danilenko, Juliya Bulanovich, Vasiliy Marchenko, Alexander Ryzhikov
EPI1813177	Russian Federation	2020 Aug 17	EPI_ISL_644129	A/goose/Omsk/0115/2020	State Research Center of Virology and Biotechnology (VECTOR)	State Research Center of Virology and Biotechnology (VECTOR)	Natalia Goncharova, Ivan Susloparov, Natalia Kolosova, Alexey Danilenko, Juliya Bulanovich, Vasiliy Marchenko, Alexander Ryzhikov

Segment ID	Country	Collection date	Isolate-ID	Isolate name	Originating Laboratory	Submitting Laboratory	Authors
EPI1813169	Russian Federation	2020 Aug 17	EPI_ISL_644128	A/goose/Omsk/0114/2020	State Research Center of Virology and Biotechnology (VECTOR)	State Research Center of Virology and Biotechnology (VECTOR)	Natalia Goncharova, Ivan Susloparov, Natalia Kolosova, Alexey Danilenko, Juliya Bulanovich, Vasiliy Marchenko, Alexander Ryzhikov
EPI1813161	Russian Federation	2020 Aug 17	EPI_ISL_644127	A/goose/Omsk/0113/2020	State Research Center of Virology and Biotechnology (VECTOR)	State Research Center of Virology and Biotechnology (VECTOR)	Natalia Goncharova, Ivan Susloparov, Natalia Kolosova, Alexey Danilenko, Juliya Bulanovich, Vasiliy Marchenko, Alexander Ryzhikov
EPI1813145	Russian Federation	2020 Aug 17	EPI_ISL_644125	A/goose/Omsk/0111/2020	State Research Center of Virology and Biotechnology (VECTOR)	State Research Center of Virology and Biotechnology (VECTOR)	Natalia Goncharova, Ivan Susloparov, Natalia Kolosova, Alexey Danilenko, Juliya Bulanovich, Vasiliy Marchenko, Alexander Ryzhikov
EPI1813121	Russian Federation	2020 Aug 13	EPI_ISL_644122	A/goose/Omsk/0002/2020	State Research Center of Virology and Biotechnology (VECTOR)	State Research Center of Virology and Biotechnology (VECTOR)	Natalia Goncharova, Ivan Susloparov, Natalia Kolosova, Alexey Danilenko, Juliya Bulanovich, Vasiliy Marchenko, Alexander Ryzhikov
EPI1814675	Belgium	2020 Nov 7	EPI_ISL_661313	A/Anser_albifrons/Belgium/11956_005/2020	Sciensano - Animal Infectious Diseases	Sciensano, Department of Animal Infectious Diseases	Steven Van Borm, Elisabeth Mathijs, Frank Vandenbussche, Thierry van den Berg, Bénédicte Lambrecht, Mieke Steensels
EPI1813417	Russian Federation	2020 Aug 17	EPI_ISL_644159	A/duck/Omsk/0076/2020	State Research Center of Virology and Biotechnology (VECTOR)	State Research Center of Virology and Biotechnology (VECTOR)	Natalia Goncharova, Ivan Susloparov, Natalia Kolosova, Alexey Danilenko, Juliya Bulanovich, Vasiliy Marchenko, Alexander Ryzhikov
EPI1813337	Russian Federation	2020 Aug 13	EPI_ISL_644149	A/duck/Omsk/0004/2020	State Research Center of Virology and Biotechnology (VECTOR)	State Research Center of Virology and Biotechnology (VECTOR)	Natalia Goncharova, Ivan Susloparov, Natalia Kolosova, Alexey Danilenko, Juliya Bulanovich, Vasiliy Marchenko, Alexander Ryzhikov

Segment ID	Country	Collection date	Isolate-ID	Isolate name	Originating Laboratory	Submitting Laboratory	Authors
EPI1813297	Russian Federation	2020 Aug 17	EPI_ISL_644144	A/duck/Omsk/0077/2020	State Research Center of Virology and Biotechnology (VECTOR)	State Research Center of Virology and Biotechnology (VECTOR)	Natalia Goncharova, Ivan Susloparov, Natalia Kolosova, Alexey Danilenko, Juliya Bulanovich, Vasiliy Marchenko, Alexander Ryzhikov
EPI1813289	Russian Federation	2020 Aug 17	EPI_ISL_644143	A/duck/Omsk/0076/2020	State Research Center of Virology and Biotechnology (VECTOR)	State Research Center of Virology and Biotechnology (VECTOR)	Natalia Goncharova, Ivan Susloparov, Natalia Kolosova, Alexey Danilenko, Juliya Bulanovich, Vasiliy Marchenko, Alexander Ryzhikov
EPI1813281	Russian Federation	2020 Aug 17	EPI_ISL_644142	A/duck/Omsk/0075/2020	State Research Center of Virology and Biotechnology (VECTOR)	State Research Center of Virology and Biotechnology (VECTOR)	Natalia Goncharova, Ivan Susloparov, Natalia Kolosova, Alexey Danilenko, Juliya Bulanovich, Vasiliy Marchenko, Alexander Ryzhikov
EPI1813137	Russian Federation	2020 Aug 13	EPI_ISL_644124	A/duck/Omsk/0004/2020	State Research Center of Virology and Biotechnology (VECTOR)	State Research Center of Virology and Biotechnology (VECTOR)	Natalia Goncharova, Ivan Susloparov, Natalia Kolosova, Alexey Danilenko, Juliya Bulanovich, Vasiliy Marchenko, Alexander Ryzhikov
EPI1812375	Netherlands	2020 Nov 2	EPI_ISL_632315	A/eurasian_teal/Netherlands/ 20016896–013/2020	Wageningen Bioveterinary Research	Wageningen Bioveterinary Research	Nancy Beerens, Frank Harders, Sylvia Verschuren-Pritz, Marit Roose, Evelien Germeraad, Marc Engelsma, Alex Bossers, Rene Heutink
EPI1813129	Russian Federation	2020 Aug 13	EPI_ISL_644123	A/turkey/Omsk/0003/2020	State Research Center of Virology and Biotechnology (VECTOR)	State Research Center of Virology and Biotechnology (VECTOR)	Natalia Goncharova, Ivan Susloparov, Natalia Kolosova, Alexey Danilenko, Juliya Bulanovich, Vasiliy Marchenko, Alexander Ryzhikov
EPI1813113	Russian Federation	2020 Aug 13	EPI_ISL_644121	A/turkey/Omsk/0001/2020	State Research Center of Virology and Biotechnology (VECTOR)	State Research Center of Virology and Biotechnology (VECTOR)	Natalia Goncharova, Ivan Susloparov, Natalia Kolosova, Alexey Danilenko, Juliya Bulanovich, Vasiliy Marchenko, Alexander Ryzhikov

Segment ID	Country	Collection date	Isolate-ID	Isolate name	Originating Laboratory	Submitting Laboratory	Authors
EPI1813830	Sweden	2020 Nov 13	EPI_ISL_647969	A/Turkey/Sweden/SVA201114SZ0001/20KN303106/2020	National Veterinary Institute, SVA	National Veterinary Institute	

*GISAID, https://www.gisaid.org.
